# False memory for orthographically versus semantically similar words in adolescents with dyslexia: a fuzzy-trace theory perspective

**DOI:** 10.1007/s11881-017-0146-6

**Published:** 2017-11-13

**Authors:** Michał Obidziński, Marek Nieznański

**Affiliations:** Institute of Psychology, Cardinal Stefan Wyszyński University, ul. Wóycickiego 1/3 bud. 14, 01-938 Warsaw, Poland

**Keywords:** Developmental dyslexia, False memory, Fuzzy-trace theory, Gist, Specific learning disorders, Verbatim

## Abstract

The presented research was conducted in order to investigate the connections between developmental dyslexia and the functioning of verbatim and gist memory traces—assumed in the fuzzy-trace theory. The participants were 71 high school students (33 with dyslexia and 38 without learning difficulties). The modified procedure and multinomial model of Stahl and Klauer (simplified conjoint recognition model) was used to collect and analyze data. Results showed statistically significant differences in four of the model parameters: (a) the probability of verbatim trace recollection upon presentation of orthographically similar stimulus was higher in the control than dyslexia group, (b) the probability of verbatim trace recollection upon presentation of semantically similar stimulus was higher in the control than dyslexia group, (c) the probability of gist trace retrieval upon presentation of semantically similar stimulus was higher in the dyslexia than control group, and (d) the probability of gist trace retrieval upon target stimulus presentation (in the semantic condition) was higher in the control than dyslexia group. The obtained results suggest differences of memory functioning in terms of verbatim and gist trace retrieval between people with and without dyslexia on specific, elementary cognitive processes postulated by the fuzzy-trace theory. These can indicate new approaches in the education of persons with developmental dyslexia, focused on specific impairments and the strengths of their memory functioning.

Over the years, studies on memory functioning in dyslexia have been conducted in various methodological and theoretical paradigms. The relationship between dyslexia and working memory (especially its phonological component) is the best studied aspect of memory functioning in dyslexia (e.g., Giofrè, Stoppa, Ferioli, Pezzuti, & Cornoldi, 2016; Kramer, Knee, & Delis, 2000; Nicolson, Fawcett, & Baddeley, 1992; Sheng, Byrd, McGregor, Zimmerman, & Bludau, 2015; Smith-Spark & Fisk, 2007). Among other memory theories that have been used in studies on dyslexia, the fuzzy-trace theory (FTT) appears to be a very promising approach to study and explain the symptoms of developmental dyslexia.

Created and actively developed by Brainerd and Reyna (i.e., 1990, 2004, 2005; Reyna, 2012) and their collaborators, the FTT has provided a comprehensive framework for research on well known effects from a novel perspective (e.g., studies on framing effect or false memories: Reyna, 2012). The FTT is based on the assumption that the process of encoding of information creates not one, but two parallel and independent memory traces representing the same stimuli. These two memory traces are the *verbatim* trace and the *gist* trace. The first is a symbolic representation of given information/stimuli, a memory trace of the surface form. It means that it encodes information like, for example, the font color, the exact character order in a word, or the exact number order in a telephone number. Verbatim trace is a precise and concrete representation of a stimulus based on its perceptual characteristics. This trace is especially important in tasks that require a high level of precision to succeed, like calculations or dialing a telephone number.

The second kind of memory representation, the gist trace, is a mental representation of information/stimuli meaning—that is, semantic properties. It means that the gist trace encodes, for example, the meaning of the words/sentences or whether a given number was rather a small or high one. Because of this, gist trace is less precise then verbatim trace but allows a person to understand cultural symbols, metaphors, etc. People use it in communication and in order to make the reasoning process more effective and less cognitively effortful. Gist trace is also more durable then verbatim trace, which means that with time, our memory of verbatim information becomes weaker than our memory of the gist trace, which leads to greater reliance on the gist trace. As already mentioned, the two memory traces are encoded in parallel, which means that the encoding of traces occurs at the same time and independently. This independence is the major difference between the FTT and the psycholinguistic understanding of the nature of verbatim and gist traces from which verbatim versus gist distinction comes from (see Reyna, 2012). Psycholinguistic theory assumes that gist develops from verbatim and, in this case, depends on it. However, studies conducted from the FTT perspective have shown that there is no need of encoding one of the traces in order to encode the other. Because of this, gist trace can be encoded without verbatim encoding (e.g., in the case of the priming effect—gist information affects our cognition without reception of any verbatim data) and vice versa, verbatim trace can be encoded with a lack of gist encoding (e.g., in the case of nonsense syllable learning).

In fuzzy-trace theory literature, we can also find assumptions about some processes involved in memory retrieval. Two of them—phantom recollection and recollection rejection—are of special interest in the context of false memory study (Brainerd, Reyna, Wright, & Mojardin, 2003; Brainerd, Wright, Reyna, & Mojardin, 2001; Stahl & Klauer, 2008, 2009). False recognition of stimuli takes place in the following cases: (1) when a retrieval cue evokes the retrieval of a gist trace but does not evoke verbatim trace retrieval, (2) when a subject is not sure about the stimulus status and makes a guess, and (3) in the case of phantom recollection, that is, due to a memory illusion based on a strong gist memory. Phantom recollection may occur when a retrieval cue is a new but very similar stimulus to the stimulus seen before; therefore, this related distracter shares the gist with a target. For example, someone may falsely accept the novel word *ocean* because it shares some meaning with the target word *sea*. However, the recollection rejection process may reduce the occurrence of false recognition and, if this is the case, the related distracter leads to target recollection and distracter rejection. For instance, if someone retrieves the verbatim trace of the target word *sea* she/he will reject *ocean* as a similar distracter.

The pattern of memory functioning in dyslexia, if we would like to describe it in terms of the FTT on the basis of previous studies, seems to be rather unclear. On the one hand, the results suggest that dyslexia is connected with an impairment of verbatim trace memory. Lots of research has shown that verbal short-term memory (STM) is impaired in people with dyslexia (e.g., Bauer, 1977; Cornwall, 1992; Kramer et al., 2000). Studies that have used procedures like *letter*-, *word*-, or *digit*-*span* (where participants have to recall a list of items in the order of their presentation) have shown that there is a significant impairment of verbal memory both in children and adults with dyslexia. Memory of the exact verbal form of the stimuli required for good performance in span tests seems to be based on verbatim trace retrieval.

It seems that various aspects of STM are impaired in people with dyslexia. In the study conducted by Smith-Spark and Fisk (2007), participants performed not only verbal but also visuospatial memory tests. There were three simple span tasks for verbal memory and one simple task for visuospatial WM, that is, the Corsi Block Span Task (where participants have to remember the location of the presented squares filled with “X” signs and recall them in the right order after each trial). In the study, there were also three complex span tasks measuring WM capacity: reading span, computation span, and spatial working memory span. Statistically significant differences in the memory performance of participants with dyslexia were observed for all the tasks, however, for the Corsi block span task, the significance was on marginal level (*p* = 0.053). These results show that the impairment of memory in dyslexia is not only connected with simple verbal tasks.

In a study which used the Hebb learning task, Bogaerts, Szmalec, Hachmann, Page, and Duyck (2015) showed how performance on the serial-order learning task is connected with dyslexia. Participants were asked to repeat—immediately after presentation—the blocks of nine consonant-vowel syllables (that were grouped into three pseudowords). One trial made use of many blocks, but one block was repeated many times throughout the whole trial. This repeated block is called the Hebb sequence. In the experiment, there were two parts of the learning phase with two separate Hebb lists. Researchers assessed how effective the learning of Hebb lists was immediately after the initial learning session and after the second and third sessions—conducted after 24 h and after a 1-month delay. The findings of this experiment showed that serial-order learning in people with dyslexia is impaired in comparison to the control group.

A study conducted by Miles, Thierry, Roberts, and Schiffeldrin (2006) examined directly how verbatim and gist trace recall works in dyslexia. In this study, participants were asked to repeat a set of sentences of varying complexity—from short and easy to complex. Researchers examined how many and what type of errors the participants had made. Their results corroborate the hypothesis that memory of verbatim trace is impaired in students with dyslexia. In comparison with typical learning students, people with dyslexia committed more errors connected with verbatim information but preserved the gist of the sentences.

On the other hand, some studies on memory functioning in dyslexia suggested that people with dyslexia differ in terms of *gist trace* memory in comparison with typically developing peers. However, the pattern of difference is not clear. There are definitions of dyslexia (e.g., Fletcher et al., 2002) that explicitly assume an impairment of gist memory functioning, which is manifested in the impairment of reading comprehension. The results of an experiment conducted by Brainerd, Forrest, Karibian, and Reyna (2006), in which the performance in the DRM task was compared between learning disabled and nondisabled children, correspond with this assumption. However, the presented studies of other investigators suggest the opposite pattern of connections between developmental dyslexia and gist memory. This research (e.g., Blau, 2013; Miles et al., 2006) showed *better* gist memory in the group of people with dyslexia in comparison with the control group. For example, in the study of Miles et al. (2006), participants with developmental dyslexia made errors and used some answering strategies connected with gist trace more often than their peers without specific learning disorders—for example, they used a synonymous word in the place of the exact word more often when they failed to recall the form of the sentence they had to repeat.

In another study, Blau (2013) used the DRM (Deese–Roediger–McDermott paradigm) task (Roediger & McDermott, 1995)—a well known praradigm, which allows researchers to evoke and examine false memories of critical words—these words are not presented on the study list during the learning phase of a memory experiment but are semantically connected with all the words from the study lists. This procedure creates a strong false memory of the critical word occurrence during the study (the probability of recall/recognition of the critical word as a word from the study list is nearly as high as the probability of a correct answer for targets). In terms of the FTT, accurate recognition is based on the retrieval of verbatim trace, while false recognition of critical words should be based on gist trace retrieval (cf., Brainerd & Reyna, 2002). The results of Blau’s (2013) study showed that participants with uncompensated language and learning disorders (LLD) rely more on gist trace retrieval then persons without LLD and those with compensated LLD—they assert that the critical word was present on the study list more often than the participants from the two other groups. There was no difference in the memory of targets, which suggests similar reliance on verbatim trace retrieval.

Nevertheless, most studies on memory functioning in dyslexia referring to the FTT (e.g., Blau, 2013; Brainerd et al., 2006; Miles et al., 2006; Voss, 2013) did not use any process-based modeling analyses. However, there are methods available that allow for process-based analysis and are adapted for the FTT—i.e., multinomial processing tree (MPT) models (e.g., Erdfelder et al., 2009). This method brings new methodological possibilities into studies of fuzzy-trace memory functioning in dyslexia. “MPT models address categorical data based on the assumption that the sample frequencies observed for a well-defined set of responses follow a multinomial distribution “(Erdfelder et al., 2009 p. 108; Riefer & Batchelder, 1988; Stahl & Klauer, 2008, 2009). This means that when there is a finite and fixed set of possible reactions on certain stimuli, the probability of every alternative reaction always sums up to 1.

A particular MPT model (adapted to a specific theory) depicts the hypothetical processes assumed in the theoretical framework, defining the set of parameters and equations—branches of the MPT model that present every possible sequence of processes leading to a particular reaction to a specified stimulus. For every kind of stimulus, there is a specific tree (the set of equations taken from the model’s branches) that describes the processes assumed by the applied theory, which can lead to every possible answer. MPT models allow researchers to study latent processes underlying overt reactions; therefore, this analysis is conducted on a more elementary level than the standard methods used in cognitive psychology research on memory in dyslexia.

The MPT model, based on the fuzzy-trace theory, was created by Brainerd, Reyna, & Mojardin, (1999) for the conjoint recognition paradigm. This paradigm refers to the distinction between the identity and similarity of a stimuli. The test list includes three types of stimuli: identical to targets, related (similar in meaning) to targets, and unrelated, novel items. In the first type of testing procedure, participants only have to accept the stimuli that are identical to the targets and reject similar and novel ones; in the second test condition, they only have to accept similar targets and reject all the other items; and in the third condition, participants have to accept both identical and similar stimuli and reject novel items. A potential difficulty of the conjoint recognition paradigm is the fact that this experimental procedure is not economical in terms of time and resources for the researcher wanting to use it in her/his studies. However, Stahl and Klauer (2008, 2009) have prepared a more convenient version of the model—the simplified conjoint recognition paradigm. In contrast to the original method, the experimental procedure includes only one test condition in which the participants receive three answer options: they recognize if the test item is the same as the target, whether it is new but related to the target, or if it is novel (i.e., new and not similar).

In the presented experiment, a simple false memory for the occurrence procedure was used (e.g., Brainerd, Gomes, & Moran, 2014; Brainerd et al., 2003; Nieznański & Tkaczyk, 2017, Stahl & Klauer, 2008) in which participants study a list of unrelated words and, during the recognition test, are presented with targets, unrelated distracters, as well as semantically (e.g., *sofa* when *couch* was the target) or orthographically (e.g., *sofa* when *soda* was the target) related distracters. This extension of the original procedure (Stahl & Klauer, 2008, 2009) allows the effect of perceptual (typo-graphical) similarity on memory functioning of dyslectic participants to be studied. This extension implies some differences in the assumptions concerning the parameters of the processing trees for the two types of distracters. The general model applied in the study contains one set of seven parameters for the semantic similarity condition and another set of seven parameters for the orthographic similarity condition.

## Method

### Participants

Seventy-one adolescents 15 to 19 years of age (*M* = 16.6 years) participated in the study; all of them were high school students. Thirty-three of the participants were students with dyslexia (14 male) and 38 were students without any learning disorders (22 male). The assignment to the first group was based on the formal diagnosis of dyslexia as documented by school files. Each participant from the control group declared that she or he has never suffered from any learning disorders.

### Material and procedure

The material used in the experiment consisted of two sets of 48 triads (each including a target word, its synonymous and orthographically similar word, e.g., *sofa* - *couch* - *soda* or *sword* - *blade* - *sworn*) and two sets of 24 unrelated words. During the study, in each condition, 54 target words were presented, among which three words were added to the study list as a primacy buffer, and three words were added as a recency buffer. In the “semantic condition,” the test list consisted of 24 targets, 24 related synonyms, and 24 unrelated distracters. In the “orthographic condition,” the test list was made up of other 24 targets, 24 orthographically similar words, and 24 unrelated distracters. In each condition, two test list versions were prepared so that if a study word appeared as a target in the first version, it was replaced by its related word in the second version and vice versa. Each version was assigned to approximately an equal number of participants.

The participants were tested in small groups, on personal computers, in a computer classroom. The presentation of stimuli materials and response recording were controlled using the E-Prime program 2.0. Written instructions were presented on computer screens and the participants started the task once they became familiar with the instructions. The study words were presented in random order at a rate of 3 s on a 19-in. computer screen. Words were printed in capital letters, Times New Roman font, with a 40-point font size. When the study list was complete, the participants solved arithmetic problems for several minutes as a buffer task. At the test phase, the participants were instructed to recognize if the presented word was the same as a target presented at study, similar to a target word or completely new (unrelated to any target). The participants were given an example of a similar word (semantically or orthographically dependent on the experimental condition). During the presentation of each test item, the response options were presented at the bottom of the screen, reminding the participants which key refers to a particular response.

Experimental conditions (semantic versus orthographic similarity) were manipulated between lists but within participants. That is, each participant took part in both conditions, half of them started from the orthographic condition and the other half from the semantic condition. The item types (target vs. related vs. novel) were manipulated within participants and lists. The dependent variables constituted the response frequencies for every kind of test stimuli and the parameters of the multinomial model measuring verbatim and gist memory processes, and response biases.

#### Multinomial model

**Fig. 1:**
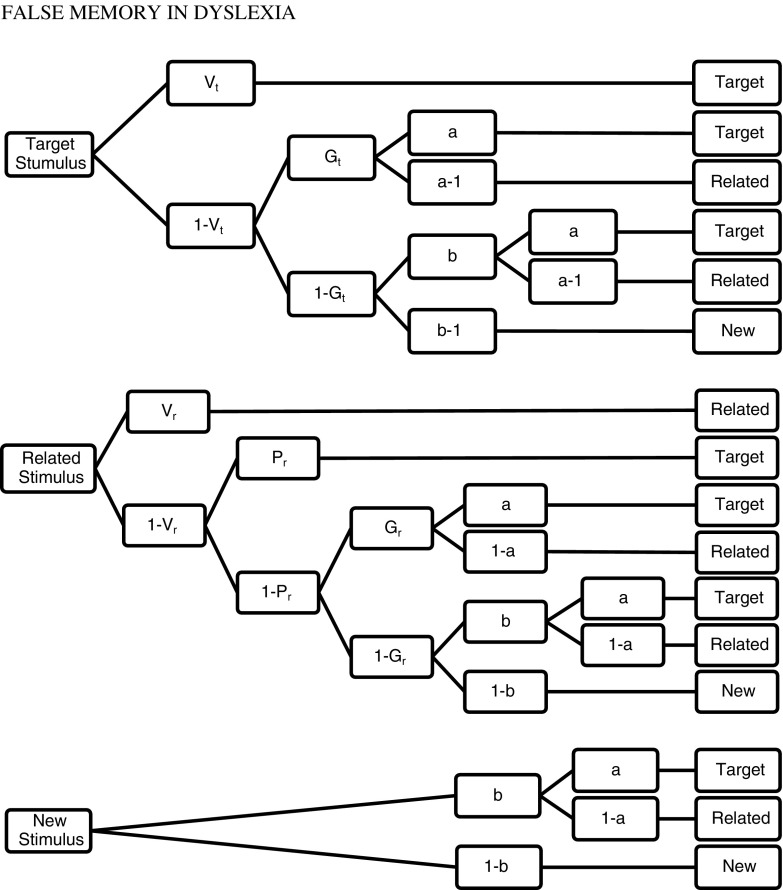
Stahl and Klauer (2009) simplified model of conjoint recognition

As the measurement model, we used the Stahl and Klauer (2008, 2009) multinomial model for the simplified conjoint recognition paradigm. However, as mentioned earlier, we extended the basic procedure by adding a new type of similar distracters. Apart from related distracters that are semantically similar to the targets (i.e., sharing the gist), we also included distracters that are orthographically similar. In the model, there are three processing trees—each for a different type of stimuli—and seven distinct parameters representing latent memory and decision processes. These processes are defined according to the FTT and represent the probability of retrieving the verbatim trace when a target is presented at test (*Vt*), as well as the probability of retrieving the verbatim trace when a related distracter is presented at test (i.e., *recollection rejection*) (*Vr*). Given no verbatim memory but available gist memory—the probability of retrieving the gist trace is represented by parameter *Gt* or parameter *Gr* when a target or related distracter is presented at test, respectively. *Phantom recollection* (*Pr*) occurs for related distracters in the absence of recollection rejection and leads to a false “target” response. The two relevant guessing processes are represented by parameters *a* and *b*. Given that no verbatim or gist memory is available for the test item, the observer may guess it is old with probability *b*. Then, parameter *a* is the probability of guessing that this undetected item is a target. Research conducted by the authors of this model (Stahl & Klauer, 2008, 2009) as well as other researchers (Nieznański & Tkaczyk, 2017) showed evidence for the validity of the model. The full version of the model contains seven parameters, which is too many in relation to 6 degrees of freedom in the data. Therefore, it is not mathematically identifiable and at least one restriction has to be imposed on the free parameters before the model can be applied. Figure 1 presents the simplified conjoint recognition model. On the left are the item types used at test, i.e., the targets, similar distracters, and unrelated distracters. On the right are the types of participants’ responses which are connected with the item types by the branches of the processing tree.

## Results

### Standard statistical test analysis

Before presenting the modeling analyses, we show the standard statistical analyses of differences in memory performance between the experimental and control groups across experimental conditions. First, the distribution normality was tested using the Shapiro-Wilk’s test for small samples. Depending on the normality test conclusions, we performed the Student *t* or Mann-Whitney *U* tests when comparing mean frequencies of responses types. A significant difference was observed only in the case of the “the same” answer in the orthographical condition when the related stimulus was presented to the participant. The group of students with dyslexia more frequently gave this answer than the participants without dyslexia. Analysis also showed a trend in the case of answering “new” in the orthographical condition when the presented stimulus was related to the target. The mean, mean rank, and standard deviations for all the variables are presented in Table 1, and the results of difference tests (Student *t* test or Mann-Whitney test) are presented in Table 2.

**Table 1:** Means, standard deviations, and mean ranks for answer probability—separately for experimental and control groups

	Control group			Experimental group		
	M	SD	MR	M	SD	MR
OTt	16.711	3.755		15.515	3.232	
OTr	2.658	3.096	30.11	3.576	1.838	42.79
OTu	4.632	2.972		4.909	3.106	
ORt	3.816	2.69	36.37	3.97	3.34	35.58
ORr	8.816	3.608		7.788	4.152	
ORu	11.368	3.983	34.67	12.242	4.373	37.53
OUt	3.211	2.44	39.01	2.606	2.524	32.53
OUr	3.921	3.672	32.11	5.273	3.668	40.48
OUu	16.868	4.545	37.64	16.121	4.853	34.11
STt	15.079	4.635		14.394	4.22	
STr	3.921	2.823	36.74	3.97	3.274	35.15
STu	5	3.263	35.03	5.636	3.707	37.12
SRt	3.5	2.565	37.03	3.818	2.845	36.86
SRr	8.342	3.85		8.879	4.547	
SRu	12.158	3.643		11.303	4.572	
SUt	3.526	3.951	37.03	2.667	2.407	34.82
SUr	5.368	3.389		6.273	4.375	
SUu	15.105	5.14		15.061	5.123	

*MR* mean rank, *OTt* probability of answering “the same” when target stimulus is presented in orthographic condition, *OTr* probability of answering “the same” when related stimulus is presented in orthographic condition, *OTu* probability of answering “the same” when unrelated stimulus is presented in orthographic condition, *ORt* probability of answering “similar” when target stimulus is presented in orthographic condition, *ORr* probability of answering “similar” when related stimulus is presented in orthographic condition, ORu probability of answering “similar” when unrelated stimulus is presented in orthographic condition, *OUt* probability of answering “new” when target stimulus is presented in orthographic condition, *OUr* probability of answering “new” when related stimulus is presented in orthographic condition, *OUu* probability of answering “new” when unrelated stimulus is presented in orthographic condition, *STt* probability of answering “the same” when target stimulus is presented in semantic condition, *STr* probability of answering “the same” when related stimulus is presented in semantic condition, *STu* probability of answering “the same” when unrelated stimulus is presented in semantic condition, *SRt* probability of answering “similar” when target stimulus is presented in semantic condition, *SRr* probability of answering “similar” when related stimulus is presented in semantic condition, *SRu* probability of answering “similar” when unrelated stimulus is presented in semantic condition, *SUt* probability of answering “new” when target stimulus is presented in semantic condition, *SUr* probability of answering “new” when related stimulus is presented in semantic condition, *SUu* probability of answering “new” when unrelated stimulus is presented in semantic condition

**Table 2:** Difference between group means for answer probability

	*F*	*p*	*t*/*U*	*df*	*p*
OTt	0.815	0.370	1.426	69	0.158
OTr			403		0.009**
OTu	0.22	0.882	− 0.384	69	0.702
ORt			613		0.871
ORr	1.427	0.236	1.116	69	0.268
ORu			576.5		0.559
OUt			512.5		0.181
OUr			479		0.086
OUu			564.5		0.470
STt	0.334	0.560	0.647	69	0.520
STr			599		0.745
STu			590		0.667
SRt			598.5		0.740
SRr	1.909	0.172	− 0.539	69	0.592
SRu	1.347	0.25	0.876	69	0.384
SUt			588		0.649
SUr	3.131	0.081	− 0.980	69	0.330
SUu	0.137	0.712	0.037	69	0.971

*OTt* probability of answering “the same” when target stimulus is presented in orthographic condition, *OTr* probability of answering “the same” when related stimulus is presented in orthographic condition, *OTu* probability of answering “the same” when unrelated stimulus is presented in orthographic condition, *ORt* probability of answering “similar” when target stimulus is presented in orthographic condition, *ORr* probability of answering “similar” when related stimulus is presented in orthographic condition, *ORu* probability of answering “similar” when unrelated stimulus is presented in orthographic condition, *OUt* probability of answering “new” when target stimulus is presented in orthographic condition, *OUr* probability of answering “new” when related stimulus is presented in orthographic condition, *OUu* probability of answering “new” when unrelated stimulus is presented in orthographic condition, *STt* probability of answering “the same” when target stimulus is presented in semantic condition, *STr* probability of answering “the same” when related stimulus is presented in semantic condition, *STu* probability of answering “the same” when unrelated stimulus is presented in semantic condition, *SRt* probability of answering “similar” when target stimulus is presented in semantic condition, *SRr* probability of answering “similar” when related stimulus is presented in semantic condition, *SRu* probability of answering “similar” when unrelated stimulus is presented in semantic condition, *SUt* probability of answering “new” when target stimulus is presented in semantic condition, *SUr* probability of answering “new” when related stimulus is presented in semantic condition, *SUu* probability of answering “new” when unrelated stimulus is presented in semantic condition

**p<0.01

### Results of MPT analyses

The use of the model requires the estimation of its goodness-of-fit assessing how well the model fits the data collected in the study. To examine the goodness-of-fit, *G* ^2^ tests are used. The results of data analysis showed that the applied model fits the collected data well, *G* ^2^ (*df* = 7) = 6.218, *p* = 0.515, and can be used for parameter estimation. To avoid saturation of the given MPT model, an assumption about parameters which can increase the number of degrees of freedom in the data has to be made. These assumptions have to be justified by theoretical or/and empirical evidences. Below, all the assumptions about the nature of some hypothetical memory processes used in this study are listed:

1. The probability of retrieving the gist trace in the orthographic condition when the presented stimulus was related to the target (*Gr*_*o*_) equals zero for both experimental groups.
2. The probability of retrieving the gist trace in the orthographic condition when the presented stimulus was the target (*Gt*_*o*_) equals zero in both experimental groups.
3. The probability of *phantom recollection* (*Pr*) for both groups equals zero.
4. The probability of retrieving the verbatim trace in the orthographic condition (*Vt*_*o*_) is the same for the control and the experimental group.
5. The probability of retrieving the verbatim trace in the semantic condition (*Vt*_*s*_) is equal for the control and the experimental group.
6. The probability of guessing “the same” answer for the experimental group is equal in both experimental conditions—orthographic and semantic (*a*_*s*_ and *a*_*o*_).
7. The probability of guessing “the same” answer for the control group is equal in both experimental conditions—orthographic and semantic (*a*_*s*_ and *a*_*o*_).
8. The probability of guessing that the new stimulus (unrelated distracter) was presented in the study phase in the orthographic condition (*b*_*o*_) is equal for the control and the experimental group.
9. The probability of guessing that the new stimulus was presented in the study phase in the semantic condition (*b*_*s*_) is equal for the control and the experimental group.

The estimators of model parameters for both the experimental and the control group and their standard errors are presented in Table 3. The difference in memory functioning between the groups was examined with the use of the *G* ^2^ statistic to check if taking the assumption about the lack of difference between given parameters significantly influences the model fit.

**Table 3:** Estimates and standard errors of used MPT model parameters probability

	Control group		Experimental group	
	P	SE	P	SE
*Gr*_*o*_	0 (constants)		0 (constants)	
*Gr*_*s*_	0.053	0.045	0.237	0.050
*Gt*_*o*_	(equal to ex. group)		0.285	0.035
*Gt*_*s*_	0.320	0.045	0.217	0.048
*Pr*_*o*_	0.090	0.020	0.112	0.019
*Pr*_*s*_	0 (constants)		0 (constants)	
*Vr*_*o*_	0.243	0.021	0.165	0.023
*Vr*_*s*_	0.149	0.030	0.018	0.050
*Vt*_*o*_	(equal to ex. group)		0.597	0.015
*Vt*_*s*_	(equal to ex. group)		0.517	0.017
*a*_*o*_	0.423	0.019	0.311	0.019
*a*_*s*_	(equal to ao)		(equal to ao)	
*b*_*o*_	(equal to ex. group)		0.312	0.011
*b*_*s*_	(equal to ex. group)		0.371	0.013

*P* probability; *SE* standard error; *Gr*_*o*_ retrieval of gist trace in orthographic condition, when related stimulus is presented; *Gr*_*s*_ retrieval of gist trace in semantic condition, when related stimulus is presented; *Gt*_*o*_ retrieval of gist trace in orthographic condition, when target stimulus is presented; *Gt*_*s*_ retrieval of gist trace in semantic condition, when target stimulus is presented; *Pr*_*o*_ phantom recollection in orthographic condition; *Pr*_*s*_ phantom recollection in semantic condition; *Vr*_*o*_ retrieval of verbatim trace in orthographic condition, when related stimulus is presented; *Vr*_*s*_ retrieval of verbatim trace in semantic condition, when related stimulus is presented; *Vt*_*o*_ retrieval of verbatim trace in orthographic condition, when target stimulus is presented; *Vt*_*s*_ retrieval of verbatim trace in semantic condition, when target stimulus is presented; *a*_*o*_ guessing “the same” in orthographic condition; *a*_*s*_ guessing “the same” in semantic condition; *b*_*o*_ guessing either “the same” or “similar” in orthographic condition; *b*_*s*_ guessing either “the same” or “similar” in semantic condition

Significant differences were observed in the case of four parameters, two connected with the verbatim trace and two related to the gist trace. The first of the differences concerned the *Gt*_*s*_ parameter (retrieving the gist when the presented stimulus is the target, the semantic condition), in which the probability of retrieval was higher for the control (0.32) than the experimental (0.217) group, *G* ^2^ (*df* = 1) = 5.183, *p* = 0.002. In contrast, in the case of retrieving gist trace, when the presented stimulus is related to the target (in the semantic condition): *Gr*_*s*_ was higher in the experimental (0.237) than the control (0.053) group, *G* ^2^ (*df* = 1) = 8.660, *p* = 0.002. The third of the parameters the value of which is significantly different between groups is the probability of retrieving the verbatim trace when the stimulus is related to target (recollection rejection) in the orthographical condition (*Vr*_*o*_). The participants without dyslexia retrieved this trace with a higher probability (0.243) than the participants with dyslexia (0.165), *G* ^2^ (*df* = 1) = 7.222, *p* = 0.004. Finally, a significant difference between parameters was also observed in the case of the probability of retrieving the verbatim trace when the stimulus is related to the target in the semantic condition (*Vr*_*s*_). Similarly to *Vr*_*o*_, the *Vr*_*s*_ parameter was higher in the control group (0.149) than in the experimental group (0.018), *G* ^2^ (*df* = 1) = 5.923, *p* = 0.009.

## Discussion

In comparison to other studies on memory functioning in dyslexia which referred to the FTT (e.g., Blau, 2013; Brainerd et al., 2006; Miles et al., 2006; Voss, 2013), the present study shows a model-based examination of differences in memory processes between students with and without dyslexia. As mentioned earlier, the usage of multinomial models allows for an analysis of the basic latent processes that are assumed in the FTT. The results of previous research conducted on this topic only gives general information on verbatim and gist trace functioning, without any distinctions between specific processes. Because of this, the conclusions formed on the basis of these studies could only show the global verbatim and gist memory functioning pattern. The results of the presented study give an insight into the hypothetical latent processes underlying memory performance. Differences were found only in some of the manifestations of verbatim and gist trace retrieval while, at the same time, differences in other processes connected therewith were not observed. This means that the impairments (and/or enchantments) of memory processes are not global in terms of verbatim and gist—which was suggested by the findings of other researchers (Blau, 2013; Brainerd et al., 2006; Miles et al., 2006; Voss, 2013).

Impairments of verbatim trace retrieval are located in the same process for both the semantic and the orthographical conditions. Deficits were found in the case of the recollection rejection process, which is responsible for the rejection of a stimulus which is only related to the target presented at study. In everyday situations, this process allows to differentiate between similar objects. This ability seems crucial in reference to the deficits and difficulties affecting people with developmental dyslexia (see American Psychiatric Association, 2013; World Health Organization, 1992). Problems with literacy like, for example, the erroneous reading of a word, can be explained in terms of the FTT. As mentioned earlier, Brainerd and Reyna’s theory is closely connected to psycholinguistic theories (Reyna, 2012); therefore, our findings could easily be connected with language learning deficits. The reading of words, on its basic level, can be connected with the verbatim trace. The developed reading skill (e.g., Indrisano & Chall, 1995)—in which reading does not involve the separate analysis of every character of a word—is based on the recognition of the whole physical structure of the word while reading it. Also, in the case of the early stages of reading skill development—in the grapheme-to-phoneme conversion process—verbatim memory is probably necessary to remember which character sign refers to a particular sound. In both cases, the similarity between given a word/characters and other objects in memory could cause errors in the reading process. Therefore, the recollection rejection process seems to play crucial role in avoiding errors caused by the similarity of one word or character to another. A deficit of differentiation between objects could not only lead to reading skill but also to writing skill errors (also observed in dyslexia); for example, one can use similar characters or character sequences in the place of the correct one and make a spelling mistake (e.g., in the Polish language, the characters “ż” and “rz” are connected with the same phoneme but cannot be used interchangeably).

Recollection rejection process, or more wide verbatim trace, plays important role not only in processing orthographically similar but also semantically similar words. Synonyms are words close but not exactly the same in meaning, but different in phonetic and graphic construction (e.g., large and big). Verbatim trace of a given word plays crucial role in distinguishing between semantically similar words because it gives additional information, that are different than nuance differences between meaning of given words. Thanks to retrieval of verbatim trace memory of gist information becoming more precise and clear, which results in less number of errors based on semantically similarities.

Another hypothesis could also explain the deficits observed in the performance of reading and writing of persons with dyslexia—i.e., grapheme-to-phoneme conversion (Tholen, Weidner, Grande, Amunts, & Heim, 2011); however, this approach fails to explain the shortcomings of the recollection rejection process. In turn, the findings concerning memory functioning in dyslexia can be used to explain other such process impairments described in literature. There is also the possibility that the deficits of these different processes coexist in dyslexia and their joint impact causes the observed deficits. The memory functioning differences on a gist trace level are not as clear as the verbatim trace findings. Similarly, only two of all the varieties of the hypothetical memory processes that are based on gist differed between experimental and control groups. The probability of gist trace retrieval when the stimuli is a target in the semantic condition (*Gt*_*s*_) was higher in the control group; on the other hand, the probability of gist trace retrieval when the stimuli is related to the target in the semantic condition (*Gr*_*s*_) is lower in the control group than in the dyslexia group. The differences in the functioning of memory of this trace are not as intuitive as those in verbatim trace memory. However, the assumptions of the FTT described by the multinomial model show possible explanations of the obtained results.

A close look at impaired and enhanced processes allows the pattern, which can be connected with compensatory mechanisms responsible for overcoming problems that people with dyslexia deal with, to be identified. Verbatim trace process differences are easily to connect with the symptoms of dyslexia, i.e., literacy skill deficits. The gist trace memory functioning pattern is more complex. It cannot be connected with directly, only through verbatim processes. The observed impairment of verbatim trace retrieval suggests that people with dyslexia have problems with correctly answering questions when differentiation between target and similar information is required. Improved gist retrieval, as measured by the *Gr*_*s*_ parameter, allows a correct answer to be given and, thus, enables an adolescent with dyslexia to overcome his or her difficulties. Unfortunately, improved gist trace memory also affects the probability of a memory mistake because gist trace could either lead to right or wrong answers—the correct recognition of the target—but also to the false recognition of a related item (Brainerd et al., 2003; Stahl & Klauer, 2008, 2009). On the other hand, poor gist retrieval when the target is presented (in the semantic condition) leads to a lower probability of both the correct answer and the false recognition of a related distracter. Therefore, this second gist trace difference (in the *Gt*_*s*_ parameter) may be a response to the *Gr*_*s*_ parameter difference. The lower probability of gist retrieval in a condition that is not influenced by verbatim trace retrieval deficits decreases the overall probability of a correct response but also counteracts the negative effects of gist trace increase in the *Gr*_*s*_ parameter, thus enhancing memory differentiation.

From the other hand, there is possibility that obtain sample constitutes a large number of students with deep dyslexia. People with this type of disorder display not only deficits in accuracy and fluency but also semantic errors (e.g., Buchanan, McEwen, Westbury, & Libben, 2003). If this situation occurred, it could explain improvement in gist trace retrieval when related stimulus is presented in semantic condition—this difference could lead to more errors with basis in gist trace, and connected with impairments of recollection rejection it fits deficits pattern found in deep dyslexia. However, second difference—impairment of gist trace retrieval when target stimulus is presented in semantic condition—leads to lover probability of gist-based memory error; thus, observed differences are not precisely match to deep dyslexia symptoms.

The FTT gives also a possible answer to the inconsistency in the findings about reading comprehension performance in dyslexia. On the one hand, some theoretical conceptions and empirical findings have assumed that process of reading comprehension in people with dyslexia is impaired in comparison to typically learning individuals (e.g., Fletcher et al., 2002; Wong, Ho, Au, McBride, Ng, Yip, & Lam, 2017). On the other hand, other studies (e.g., Miles et al., 2006 and the presented study), conducted under the FTT, suggest that gist trace memory (connected with comprehension; see Weekes, Hamilton, Oakhill, & Holliday, 2008) could be the same or even better in dyslectic than in typically learning individuals. However, this inconsistency can stem from the age differences of studied groups. Research based on FTT shows that there is the swap of memory reliance from verbatim trace to gist trace (Reyna, 2012) during development. In fact, young children could rely mostly or even entirely on verbatim processing in the comprehension of a read text; thus, reading comprehension observed in studies on young children (Wong et al., 2017) could not be related with the performance of gist trace memory.

In the context of education, obtained result could suggest possible approaches in methods of teaching of adolescence with dyslexia. Firstly, teachers should remember about deficits in recollection rejection process (thus, impairments of verbatim memory) that affect pupils with dyslexia. This kind of impairment could lead to errors not only in reading but also spelling and even memory of other type, precise information—if deficits are not narrowed to verbal memory. Because of that, teachers should based their evaluations of pupils’ works more on the gist information, and less on memory of exact information (like date in history, or remembering of names and text in the language classes). In the same time, teachers should give to their pupils with dyslexia some additional exercises that focus their attention on mistakes their made and help them to remember correct forms, answers, etc. Even if deficits will affect only reading, this approach still can bring good results. From the one hand, students with dyslexia will not feel worst then other student; thus, their situation will less affect their self-esteem. But, on the other hand, they will still have opportunity to work on their mistakes to overcome their difficulties. Secondly, knowledge about which particular process are impaired should lead to more focus forms of therapy and exercises that aim to improve this specific disabilities. Results of Moore and Lampinen (2016) research suggest that people are aware of using recollection rejection in their attempt to remember something. Therefore, it is possible that someone could train this memory process to improve its use in everyday situations. However, this approach needs further investigation.

The presented studies offer new opportunities based on the FTT memory model for the examination of the connections existing between developmental dyslexia in adolescents and their memory functioning. The application of the multinomial model gave an insight into the more elementary memory processes assumed in the used theory, which allows for a deeper and more precise examination of memory functioning in dyslexia than in other studies using standard analysis methods. Nevertheless, more studies based on FTT and multinomial modeling have to be conducted to comprehensively analyze the connections between developmental dyslexia and the functioning of verbatim and gist memory. The present research only covers one age group—adolescents. There is a possibility, supported by theoretical and empirical evidence, that the pattern of memory functioning in people with dyslexia in different age groups could be different. This is precisely why future studies should compare memory functioning not only between participants with developmental dyslexia and their peers but also between participants in different age groups.
